# Automatic Intra-/Extra-Dimensional Attentional Set-Shifting Task in Adolescent Mice

**DOI:** 10.3389/fnbeh.2021.704684

**Published:** 2021-07-19

**Authors:** Mariasole Ciampoli, Diego Scheggia, Francesco Papaleo

**Affiliations:** ^1^Genetics of Cognition Laboratory, Neuroscience Area, Istituto Italiano di Tecnologia, Genoa, Italy; ^2^Department of Pharmacological and Biomolecular Sciences, University of Milan, Milan, Italy; ^3^Fondazione IRCCS Ca’ Granda Ospedale Maggiore Policlinico, Milan, Italy

**Keywords:** development, attentional control, automated task, executive function, dysbindin-1, mice, attentional set-shifting

## Abstract

Adolescence is a developmental period crucial for the maturation of higher-order cognitive functions. Indeed, adolescence deficits in executive functions are strong predictors of increased vulnerability to several mental disabilities later in life. Here, we tested adolescent mice in a fully-automated attentional set-shifting task equivalent to the humans’ Wisconsin Card Sorting Test (WCST) and the Cambridge Neuropsychological Test Automated Battery Intra-/Extra-Dimensional set-shift task (ID/ED). Compared to an adult, adolescent mice required more time to complete the task (≈16 days), and a higher percentage failed to finish the entire task. Nevertheless, adolescent mice completing this demanding task showed an increased effort in solving the extradimensional shift stage (EDS) compared to previous stages. Moreover, we found that this paradigm can be used to detect early cognitive dysfunctions in adolescent genetically modified mice. Thus, this automatic paradigm provides a further tool to assess attentional control in adolescent mice, and the development of dysfunctional executive functions from adolescence to adulthood.

## Introduction

Executive functions include the ability to monitor and flexibly change behavior when facing novel situations. These abilities develop from early childhood and mature during early adolescence and young adulthood (Barnett et al., 2010; Luna et al., 2015; Sousa et al., 2018). Significant improvements in cognitive processing speed and intellectual functioning are evident throughout late childhood and adolescence, particularly involving the development of flexible planning, decision-making, and response inhibition (Luna et al., 2004). Early maturational deficits in executive functions are often indicated as strong predictor of later disabilities or increased vulnerability to psychiatric disorders, including schizophrenia (Cannon, 2008; Rapoport et al., 2012).

Neuropsychological assessment of executive functions is commonly done in adolescents, similarly to adults, using tasks such as the Wisconsin Card Sorting Test (WCST; Hintze and Borkowska, 2015; Pontillo et al., 2019), and the CANTAB Intra-/Extra-dimensional set-shifting task (ID/ED; Barnett et al., 2010; Keeler and Robbins, 2011; Papanastasiou et al., 2018; Morrison et al., 2020). These tasks measure attentional set-shifting abilities (Keeler and Robbins, 2011) and are sensitive in detecting early cognitive abnormalities in a wide range of mental disorders including autism spectrum disorders (Hill, 2004; Kado et al., 2020), schizophrenia (Ceaser et al., 2008; Pontillo et al., 2019), obsessive-compulsive disorders (Head et al., 1989; Pauls et al., 2014) and attention-deficit/hyperactive disorders (ADHD; Chamberlain et al., 2011; Uytun et al., 2017). Cognitive alterations revealed by these tasks during adolescence are strong predictors of later life functioning and can be used to detect vulnerability by genetic and/or environmental risk factors with long-lasting effects (Casey et al., 2005; Blakemore and Choudhury, 2006; Marco et al., 2011; Dumontheil, 2016).

The WCST and CANTAB ID/ED paradigms have been successfully translated and validated also in non-human primates, rats, and mice (Birrell and Brown, 2000; Garner et al., 2006; Bissonette et al., 2008; Papaleo et al., 2008; Scheggia et al., 2012, 2018), demonstrating similar functional specializations of the underlying brain networks involved (Shepard et al., 2017). However, measurements of attentional set-shifting and executive functions in adolescent rodents are still scarce and only a few studies have investigated such cognitive functions in adolescents. The major challenges limiting the application of such complex paradigms in rodents are the short developmental period of their adolescence (≈25–50 postnatal day (PNDs), and the increased vulnerability to stressful manipulations during such period. For example, by a modification of the 5-Choice Serial Reaction Time Task (5CSRTT), we were recently able to assess attentional control and distractibility in adolescent mice, in about 10–15 days, with no interference by the experimenters during task execution (Ciampoli et al., 2017). More specific to attentional set-shifting paradigms, it has been reported that adolescent rats and mice might have more problems in solving basic compound discrimination (CD) stages as well as reversal and extra-dimensional shift (EDS) stages in a manual “digging” version of the task (Cain et al., 2011; Newman and McGaughy, 2011; Shepard et al., 2017). However, in these latter studies, the performance of adult rodents was not consistent with the development of an attentional set as previously reported (Birrell and Brown, 2000). Moreover, all of them required intense manipulations (because of the manual version), and heavy food restriction regimen (85–90%, which could be an issue during the fast growth happening during adolescence).

Here, we tested adolescent mice in an automated attentional set-shifting task well-validated for adult mice (Scheggia et al., 2014, 2018) The automated version of the task could be useful to eliminate any source of subjectivity and stressful manipulation, and avoiding arbitrary environmental conditions. Measuring executive functions in adolescent mice, strictly controlling environmental and genetic factors as well as applying advanced mechanistic manipulations, could allow testing of early interventions and treatments, which are considered of critical importance in humans psychopathology (Hadar et al., 2017; Armando et al., 2020). Thus, we first characterized the performance of adolescent mice in the Two-Chamber “Operon” ID/ED Task comparing their performance to equivalent parameters used in adult mice (Scheggia et al., 2014). To provide further validation of this task in adolescent mice, we then tested a mouse line with a genetic disruption of the Dysbindin-1 gene for which we tested adult mice in the equivalent task, and for which we have proven translational validation in healthy human subjects as well as in patients with schizophrenia (Scheggia et al., 2018; Leggio et al., 2019). We found a distinct behavioral performance of adolescent mice in the ID/ED task compared to adult mice, in agreement with the idea that executive function abilities are still developing during this period. Moreover, we provide evidence that this task can reliably detect early cognitive dysfunctions in mice, enriching the tools available to assess developmental trajectories and potential early intervention in mice. Overall, these data support the use of the automated ID/ED task for the assessment of executive (dys)functions in adolescent mice.

## Materials and Methods

### Animals

All procedures were approved by the Italian Ministry of Health (permit n. 230/2009-B) and strictly adhere to the recommendations in the Guide for the Care and Use of Laboratory Animals of the National Institutes of Health. Dysbindin-1 null mutant (DYS −/−), their heterozygous (DYS +/−) and wildtype (DYS +/+ DYS +/+) littermates on a C57BL/6J genetic background were used (Scheggia et al., 2018). A Dys+/− × Dys +/− breeding scheme was used. Mice were identified by PCR analysis of tail DNA. Mice were weaned at 28 postnatal day (PND) and tested during adolescence or adulthood. The “adolescent” time period is generally corresponding to the onset of puberty (from about 9 to 12 and 15 to 17-year-old in humans; from about 28–50 days old in rodents (Adriani and Laviola, 2003; Schneider, 2013). For adolescent mice, we used in-house bred male mice within the range of 28–50 days old. From PND 28 to PND 30 mice were singly housed, daily exposed to 1-min handling session, given ten 14-mg pellets of the 5TUL diet and weighted: during this time mice were food-restricted to maintain 95% of their baseline free-feeding body weight. Water was freely available only in the home cage. Mice were housed in a climate-controlled animal facility (21 ± 2°C) and maintained on a 12-hour light/dark cycle (light on: 7 a.m. to 7 p.m.). Testing was conducted during the light phase (10 a.m. to 5 p.m). For comparison, we selected adult mice tested in the same time period (between 2012–2014), and in the same apparatus and paradigm, extracted and combined from our previously published studies (Scheggia et al., 2014, 2018).

### Two-Chamber Operon ID/ED Task

**Figure 1:**
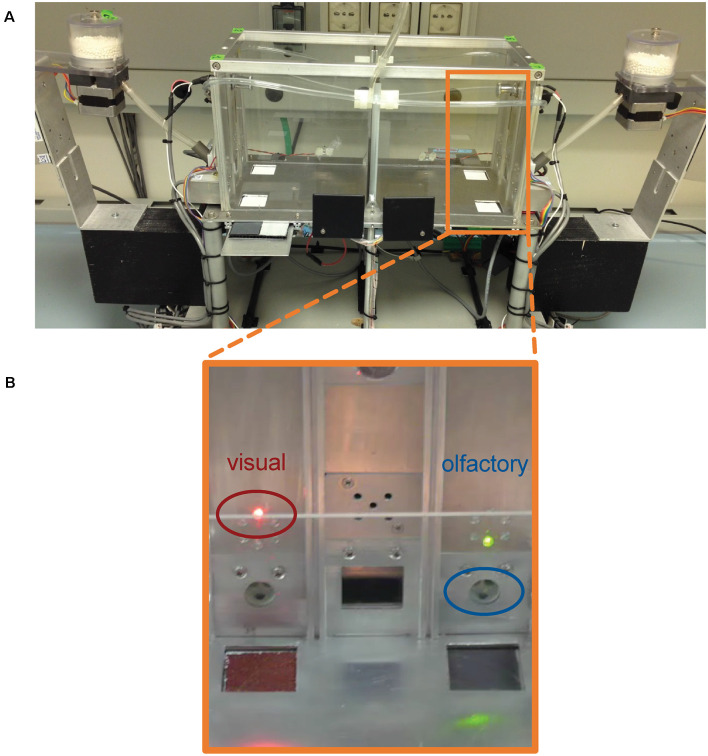
**(A)** Photo of the two-chamber “*Operon*” apparatus. **(B)** Close up of the wall containing visual and olfactory stimuli. Light stimuli are located on the top of each nose poke hole. Light stimuli were switched on in pairs (red-green, yellow-blue, white-orange). Olfactory stimuli were presented inside the nose poke holes. All stimuli were counterbalanced between left and right nose poke holes.

We used the two-chamber “Operon” ID/ED task as previously described in adult C57BL6J mice and adult dysbindin-1 +/− mice (Scheggia et al., 2014). The apparatus consisted of two identical chambers with Plexiglas walls and an aluminum floor separated by an automatic transparent door that dropped to allow the mouse to change chambers (Figure 1). The two chambers allowed us for automatic and continuous stimuli changes without interfering with the mouse. Infrared photobeams tracked the animal movements and controlled the opening and closing of the automatic door to allow the mouse to change chambers. Each chamber presented two nose-poke holes with infrared photobeams, and, between them, a food magazine with photobeams delivering 14-mg rodent pellets. A fan and a house light were located above each of the two food magazines. Each nose-poke hole was equipped with a series of changeable stimuli that could vary in two perceptual dimensions (odor or view). For olfactory stimuli, liquid odorants were diluted in mineral oil (1:20; M5904, Sigma Aldrich, Dorset, United Kingdom). For odors, two dilution olfactometers were used (PHM-275; Med Associates) that controlled the presentation of odor pairs inside the nose-poke holes. For visual stimuli, light-emitting diodes were placed on top of each nose-poke hole. Up to six color stimulus exemplars could be presented to the test subject. Light stimuli were switched on in pairs (red–green, yellow–blue, white–orange) and counterbalanced between left and right nose-poke holes. Thus, the discriminative association between a correct response (which will result in food delivery) and a nose-poke hole could be varied by their odor or visual cue. Stimuli and operant-based rewarding components were located in the same positions in chamber 1 and chamber 2. Pairs of stimuli are randomly presented in each stage, and the mouse must choose the correct stimulus in each pair. In the simple discrimination (SD) stage, the mice were introduced to a dimension (odor or light) that was relevant throughout the tasks until the extradimensional shift (EDS).

### Habituation

On the first day, mice were trained for 40 min to move inside the two-chamber Operon apparatus without the door divider, with nose-poking into any nose poke hole resulting in a pellet delivery into the food receptacle. During this phase, only neutral stimuli for all different dimensions were used (Habituation phase 1). On the second day, mice were placed for 40 min in the Operon chambers with only neutral stimuli for all different dimensions and were trained to move from one chamber to the other at the end of each trial (Habituation phase 2). A session was started by placing the mouse in one chamber with neutral stimuli cues. A free pellet was delivered and a trial began when the subject entered the food magazine. This dropped the door to give the mouse access to the other chamber. Infrared photobeams flanking the door between the two chambers detected the movements of the animal so that the door was closed when the mouse moved from one chamber to the other. A nose poke into any of the nose-poke holes resulted in a pellet delivery into the food magazine. Infrared photobeams inside the food magazine detected the mouse while it retrieved the food reward and dropped the door to give the mouse access to the other chamber for the next trial. On the 3rd day (Habituation phase 3), the mice were trained to perform two simple discriminations (SD1 and SD2; for example, light cue on vs. light cue off; peach vs. sage) to a criterion of eight correct responses out of 10 consecutive trials so that they were familiar with the stimulus dimensions. These exemplar pairs were not used again during the test. A session started by placing the mouse in one chamber with neutral stimuli, while in the other chamber the stimuli cues were turned on or placed in position. Then, the door was dropped to give the mouse access to the other chamber with the stimuli cues on and the mouse had to choose the nose-poke hole in which it was presented the correct stimulus (e.g., first yellow light and then the peach odor). Nose poking into the correct hole resulted in a pellet delivery and, when the mouse entered the food magazine, the door was dropped to give the mouse access to the other chamber for the next trial. Nose poking in the incorrect hole was not rewarded and the house light was turned on for 5 s. Following these 5 s with the house light on, the door was dropped to give access to the other chamber for the next trial. The first 10 trials of each stage were exploratory trials: if the mouse selected the incorrect hole, an error was recorded but the trial was not terminated until the mouse poked also in the correct hole. In subsequent trials, if the mouse poked in the incorrect hole, an error was recorded, the trial was terminated, and all dimensional stimuli were turned off. The apparatus was wiped down with 70% alcohol at the end of each mouse session.

### Test

We employed a ‘two-dimensional’ ID/ED paradigm as in previous studies in humans, non-human primates, and rodents (Dias et al., 1996; Birrell and Brown, 2000; Barnett et al., 2010; Scheggia et al., 2014, 2018). The task requires the rodent to learn to associate a food reward with a specific dimension which is later changed during the task. The mice will be exposed to the task with a series of discrimination that involves a simple discrimination (SD), compound discrimination (CD), compound discrimination reversal (CDRe), intra-dimensional shift (IDS), intra-dimensional reversal (IDRe), intradimensional shift 2 (IDS2), intra-dimensional shift 2 reversal (IDS2Re), extra-dimensional shift (EDS), and extra-dimensional reversal (EDRe). An example of the entire task is diagrammed in Table 1. Throughout the task, the mice will perform each stage to reach a criterion of eight correct choices out of 10 consecutive. This task allows the specific investigation of the mechanism underlying both attentional set formation and maintenance in addition to attentional flexibility and reversal learning. In the SD, the nose-poke holes differed along one of two dimensions (e.g., odor or light). For the CD, a second dimension was introduced, but the correct and incorrect exemplars remained constant. For the reversals (CDRe, IDSRe, IDS2Re, and EDSRe), the exemplars and the relevant dimension were unchanged: the animal had to learn that the previously correct stimulus was now incorrect. For the IDS, IDS2, and EDS, there were new exemplars of both the relevant and irrelevant dimensions. In the EDS stage, the previously relevant dimension was now the irrelevant dimension. The order of the discriminations was always the same, but the dimensions and the pairs of exemplars were equally represented within groups and counterbalanced between groups as far as possible. There were two possible patterns of shift: light to odor and odor to light. Mice were counterbalanced so that half of the mice received light as the initial relevant dimension and the other half odor. The side of stimulus presentation (left/right) was randomly selected by MED-PC IV software (Med Associates, St. Albans, VT). The selection and side presentation of the lights and odors were randomly determined by MED-PC IV software (Med Associates, St. Albans, VT) at the beginning of each trial. Performance was measured by: number of trials to reach the criterion; time to reach the criterion; time from breaking the photobeams adjacent to the automated dropping door to a nose-poke response (latency to respond). For each stage, we set a maximum 150-trial cut-off rule. During the habituation, mice that met these criteria were excluded and were not further tested. During the task, a stage was terminated when mice met the 150-trial/stage cut-off rule, and an attempt was made to move them on to the following stage. However, mice that reached the cut-off and did not complete more than two stages were excluded.

**Table 1:** An example of the order of discriminations presented to the mice in the attentional set shifting automated task.

Stage	Relevant dimension	Irrelevant dimension
SD	**Odor**	Light
CD	**Odor**	Light
CDR	**Odor**	Light
IDS	**Odor**	Light
IDSRe	**Odor**	Light
IDS2	**Odor**	Light
IDSRe2	**Odor**	Light
EDS	**Light**	Odor
EDSRe	**Light**	Odor

*In this example, the relevant dimension for the mouse was the odor until the EDS, in which the relevant dimension became the color of the light stimuli. The stages signify the different shifts. On each trial, the bold is the correct choice. The combination of exemplars and their left-right position of presentation in the apparatus is a pseudorandom series determined in advance.*.

### Statistics

Results are expressed as mean ± standard error of the mean (SEM) throughout the manuscript. One-way analyses of variance (ANOVAs) with the different stages (SD, CD, CDRe, IDS, IDSRe, IDS2, IDSRe2, EDS, and EDSRe) as a within-subject factor was used to examine the performance in the tasl (number of trials to reach the criteria, timing needed to complete each stage and the latency to respond). In the experiment using genetically modified mice, two-way ANOVAs with genotype (DYS +/+, +/− and −/−) as a between-subjects factor and the different stages (SD, CD, CDRe, IDS, IDSRe, IDS2, IDSRe2, EDS, and EDSRe) as a within- subject factor was used to examine the number of trials necessary to reach the criteria, timing needed to complete each stage and the latency to respond. *Post hoc* analyses were conducted using Tuckey’s multiple comparison test. The accepted value for significance was *p* < 0.05. All statistical analyses were performed using GraphPad Prism.

## Results

### Adolescent Mice Readily Acquire the Automatic Attentional Set-Shifting Task

**Figure 2:**
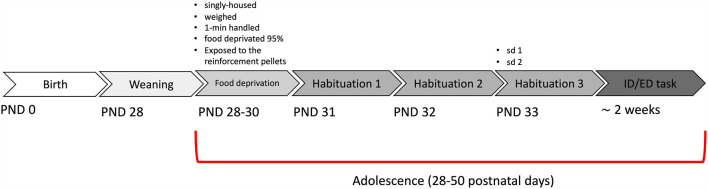
Timeline of the experiment in adolescent mice.

Mice were weaned at PND28, single-housed, and food-restricted to start the test from PND31 (Figure 2). We tested a total of 13 wild-type DYS+/+, 13 DYS +/− and nine DYS−/−. First, we analyzed the data from wild-type mice contrasting their results with equivalent parameters obtained in adult mice (Scheggia et al., 2014, 2018).

**Figure 3:**
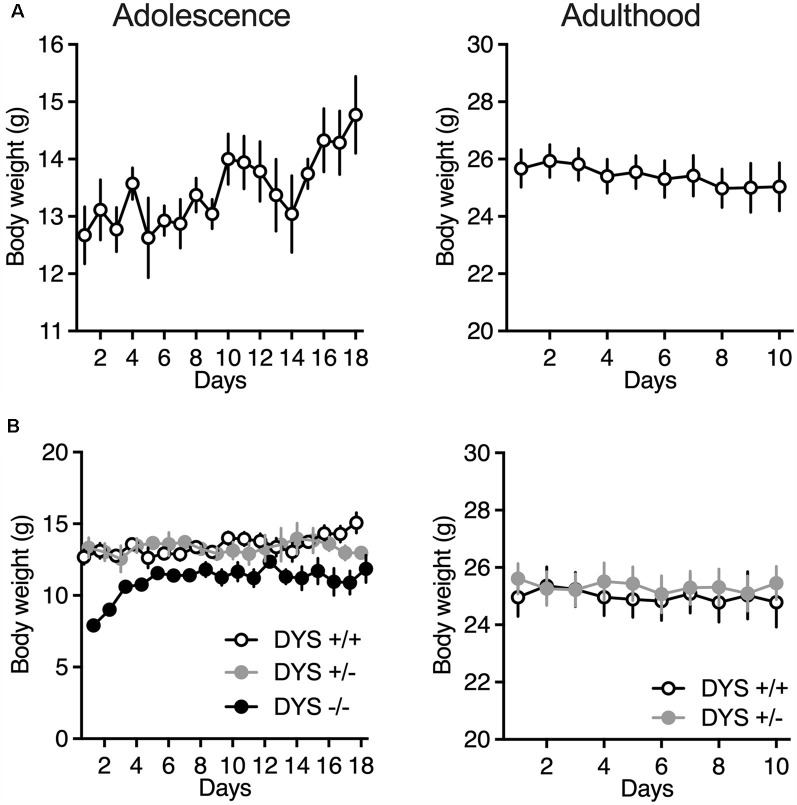
**(A)** Body weight of adolescent and adult wild-type mice during the ID/ED *Operon* task. **(B)** Body weight of adolescent DYS +/+, DYS +/−, and DYS −/− mice during the ID/ED *Operon* task. Values represent mean ± SEM. Abbreviations: ID, intra-dimensional set-shift task; ED, extra-dimensional set-shift task.

Adult mice were food-restricted throughout the experiment in order to maintain about 90% of their baseline free-feeding body weight. In adolescent mice we kept a less strict food restriction regimen of about 95% or less of their initial baseline (Figure 3A), to allow an increase in body weight throughout the days of testing.

**Figure 4:**
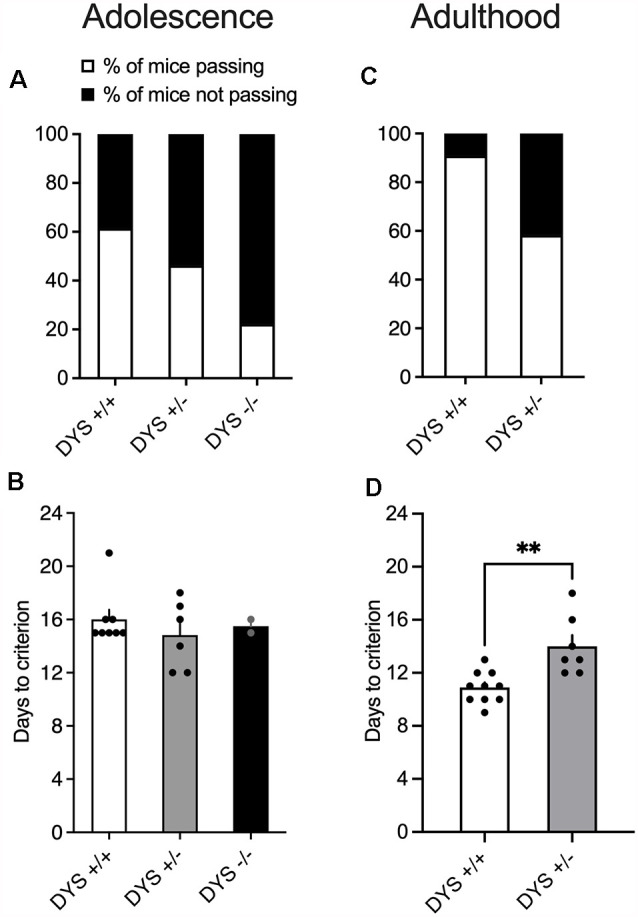
**(A)** Percentage of mice finishing the entire task. 13 adolescent DYS +/+, 13 DYS +/−, and nine DYS −/− mice were tested. Five DYS +/+, seven DYS +/−, and seven DYS −/− mice were excluded because they were not reliably poking to retrieve the food reinforcement during the training or were not able to finish the entire procedure. All the other mice readily learned to poke in the holes for food rewards and were able to perform multiple sequences of discriminations. **(B)** The number of days taken by DYS +/+, DYS +/−, DYS −/− to complete the entire procedure of the ID/ED task (only those mice that finished the entire task are depicted in this bar graph). **(C)** Percentage of adult DYS +/+, and DYS +/− mice passing the entire task. **(D)** The number of days taken by DYS +/+ and DYS +/− adult mice to complete the entire procedure of the ID/ED *Operon* task (only those mice that finished the entire task are depicted in this bar graph). Values represent mean ± SEM. DYS +/− adult mice need more days to reach the criterion compared to DYS +/+. **p < 0.005.

Out of the 13 adolescent wild-type mice tested, eight were able to complete the entire task (Figure 4A), with an average of 16 days (Figure 4B). Notably, even the slowest performing mice at the end of the test were 50 days old which is still considered late adolescence in mice (Adriani and Laviola, 2003). Excluded mice were not able to finish the entire procedure, reaching the fixed maximum cut-off trials numbers for single stage as detailed in the Methods section. In adult mice, the percentage of mice completing the test reached more than 80% (Figure 4C). Moreover, adult mice usually complete the entire task faster, in about 10 days (Figure 4D).

**Figure 5:**
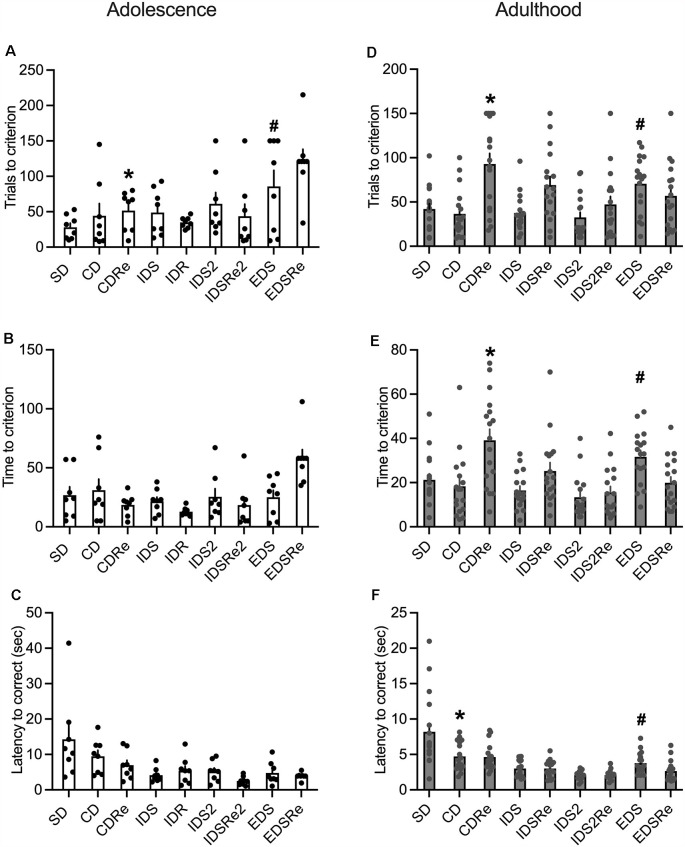
**(A)** Number of trials (*CDRe: *p* < 0.005 vs. EDSRe. ^#^EDS: *p* < 0.05 vs. SD, CD, IDR) and **(B)** time required by wild-type adolescent mice to reach the criterion in the different stages of ID/ED *Operon* task (*n* = 8). **(C)** Latency to respond in each stage of the ID/ED *Operon* task in wild-type adolescent mice. **(D)** Number of trials (*CDRe: *p* < 0.0005 vs. SD, CD, IDS and IDS2, *p* < 0.005 vs. IDS2Re, *p* < 0.05 vs. EDSRe. ^#^EDS: *p* < 0.05 vs. CD, IDS, IDS2) and **(E)** time required by wild-type adult mice to reach the criterion in the different stages of ID/ED *Operon* task (*CDRe: *p* < 0.00,005 vs. CD, *p* < 0.05 vs. IDSRe, *p* < 0.0005 vs. IDS2Re, *p* < 0.0005 vs. EDSRe. ^#^EDS: *p* < 0.05 vs. CD, IDS, *p* < 0.0005 vs. IDS2). **(F)** Latency to respond in each stage of the ID/ED *Operon* task in wild-type adult mice (*CD *p* < 0.00,005 vs. IDS2. ^#^EDS *p* < 0.0005 vs. IDS2). Values represent mean ± SEM. Abbreviations: CDRe, compound discrimination reversal; IDS2Re, intra-dimensional shift 2 reversal; SD, simple discrimination; EDSRe, extra-dimensional shift reversal.

The analysis of adolescent wild-type mice performance revealed a discrimination effect for the number of trials (*F*_8,63_ = 3.90; *p* = 0.0009), as they needed more trials to complete the EDS stage compared to SD, CD, and IDR (Figure 5A). This suggests the development of an attentional set. However, we did not observe such an effect in the time needed to complete the stages (Figure 5B). In contrast, adult mice required more trials (*F*_8,128_ = 7.25; *p* < 0.0001; Figure 5D), and more time (*F*_8,128_ = 8.15; *p* < 0.0001; Figure 5E) to solve the EDS compared with CD, IDS, IDS2, and EDSRe.

Adolescent mice did not display any significant effects on the reversal stages (Figures 5A–C), while this was evident in adult mice (Figures 5D,E). Moreover, whereas adult mice required fewer trials (Figure 5D) and less time (Figure 5E) to solve the EDSRe stage compared to the EDS, this effect was not evident in adolescent mice, which even show increased time to solve this stage compared to SD and CD (F_8,63_ = 4.16; *p* = 0.0005, Figure 5B). Finally, the analysis of the latency to respond showed in both adult (F_8,128_ = 16.74; *p* < 0.0001) and adolescent (F_8,63_ = 4.29; *p* = 0.0004) mice a decreasing duration, consistent with a better performance throughout the task (Figures 5C,F). However, while adults showed an increased latency to respond in the EDS stage compared to the previous discrimination in the IDS2 and CD stages (Figure 5F),this was not significant in adolescent mice (Figure 5C).

Overall, these data show that adolescent mice can perform the automated attentional set-shifting task, even if less efficiently than adults, showing increased effort to shift from an attentional set to another, while showing less evident vulnerability to intradimensional shifts and reversals.

### Cognitive Deficits in DYS-Deficient Adolescent Mice

To further validate the attentional set-shifting task in adolescent mice, we tested DYS mutant mice that have been previously tested in the same task during adulthood (Scheggia et al., 2018).

DYS−/− adolescent mice lost more body weight during the entire test compared to DYS +/− and DYS +/+ littermates (Figure 3B). Compared to DYS +/+ mice, a higher percentage of adolescent DYS +/− and DYS−/− littermates were not reliably poking to retrieve the food reinforcement during the training or were not able to finish the entire procedure, reaching the fixed maximum cut-off trials numbers for the single stage as detailed in the Methods section (Figure 4A). All mice were normally poking and no correlation was evident between body weight and test performance, suggesting no implication of motivation-related issues, but instead a more cognitive or poorer attentional component. This is in agreement with data obtained in adult DYS mutant mice (Figure 4C). All the other adolescent mice were able to complete the task in about 15 days with no DYS-dependent genotype effect (Figure 4B).

**Figure 6:**
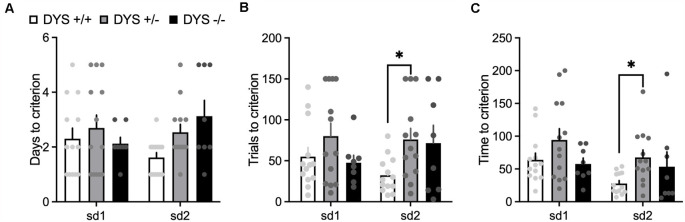
**(A)** Days **(B)** number of trials, and **(C)** time required by DYS +/+, DYS +/−, and DYS −/−, adolescent mice to reach the criterion during the simple discrimination 1 (sd1) and 2 (sd2) of the habituation (phase 3) of the ID/ED *Operon* task. *p < 0.05 vs. DYS +/+ at the same stage. Values represent mean ± SEM.

**Figure 7:**
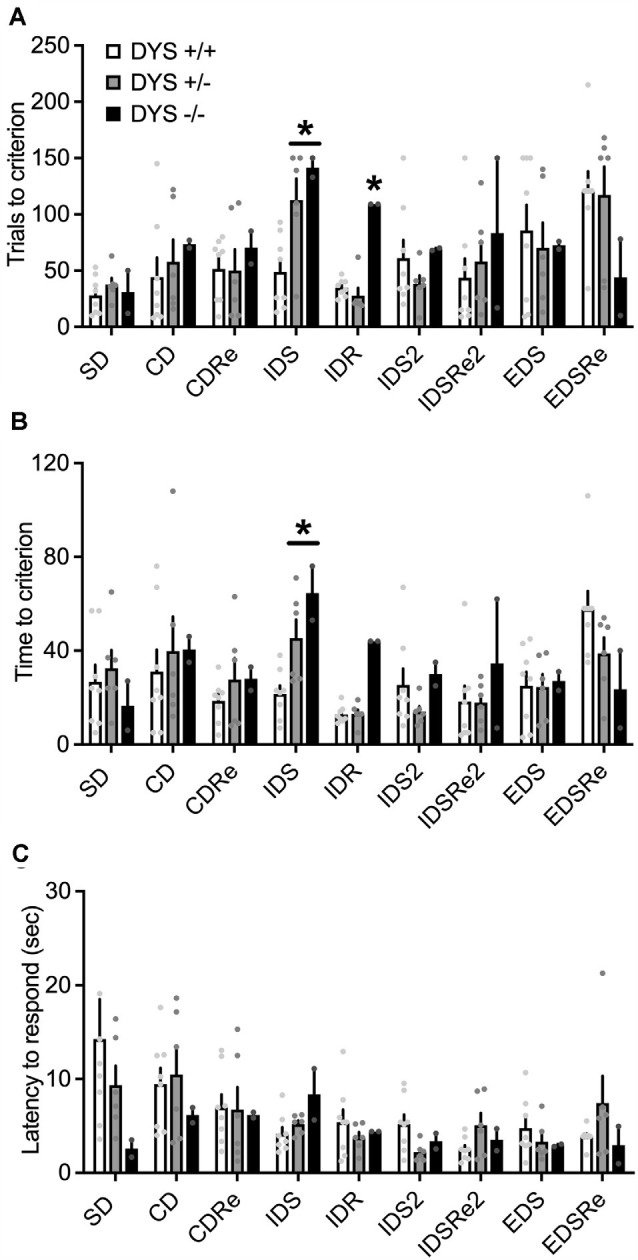
**(A)** Number of trials and **(B)** time required by DYS +/+, DYS +/−, and DYS −/− adolescent mice to reach the criterion in the different stages of, ID/ED *Operon* task. **(C)** Latency to respond in each stage of the ID/ED *Operon* task in DYS +/+, DYS +/−, and DYS −/− adolescent mice. *p < 0.05 vs. DYS +/+ mice at the same stage. Values represent mean ± SEM.

In the habituation training, consisting of two simple discriminations, no effect of DYS genotype (*F*_2,38_ = 2.20; *p* = 0.11), stage (*F*_1,62_ = 0.02; *p* = 0.87), or their interaction (*F*_2,62_ = 2.22; *p* = 0.11), was evident for the days needed to reach the criterion (Figure 6A). However, DYS +/− required more trials (genotype, *F*_2,62_ = 3.08; *p* = 0.02; Figure 6B) and time (genotype, *F*_2,62_ = 3.66; *p* = 0.03; Figure 6C) to complete SD2 stage compared with DYS +/+ mice (*p* < 0.05). During the task, we found a significant stage x genotype interaction for the trials (*F*_16,104_ = 1.67; *p* < 0.05, Figure 7A) and time (*F*_16,104_ = 1.79; *p* < 0.05, Figure 7B) needed to complete the task. In particular, *post hoc* analysis revealed that DYS +/− required more trials (*p* < 0.05; Figure 7A), and time (Figure 7B) to complete the IDS stage compared with DYS +/+ mice. Finally, the analysis of the latency to respond showed a significant effect of the stage (*F*_8,104_ = 2.50; *p* < 0.05). In particular, mice improved their speed to respond at the IDS2 stage compared with IDS in a genotype-independent way (*p* < 0.05; Figure 7C).

Overall, these experiments show that executive functions’ deficits in DYS +/− mice are already evident during adolescence.

## Discussion

In this study, we tested adolescent mice in a fully automated attentional set-shifting task previously validated in adult mice. With consistent differences compared to adults, we showed that this task could be effective for an early assessment of executive functions’ abilities and abnormalities during rodents’ adolescence.

Despite the complexity of the task, about 60% of the tested wild-type adolescent mice effectively completed the entire procedure. Only a few previous studies showed that adolescent mice (Birrell and Brown, 2000) and rats (Cain et al., 2011; Newman and McGaughy, 2011) were able to complete attentional set-shifting paradigms. The major advantage brought by our task is the automated procedure that can reduce the stressful component present in the manual version, as we previously demonstrated (Scheggia et al., 2014). Despite the duration of the procedure, this task can be completed before the end of adolescence (≈50 postnatal days) in mice. Moreover, differently from previous studies (Bizot et al., 2015; Shepard et al., 2017), here we were able to test the mice adopting a less stringent food restriction protocol, which still allows them to increase their body weight through the testing phase. This is important as adolescent rodents are rapidly growing, so a less stringent food restriction is preferable (Hueston et al., 2017). We believe that an even less strict food restriction regimen with an incremental increase of food could be even more effective in limiting the amount of stress and improve further the performance of tested mice. In our experience, a stringent food restriction can strongly impair attentional set-shifting abilities, making the mice not able to perform and finish the entire protocol. Thus, when testing adolescent mice, the combination of an automated test with very mild food restriction might provide important advantages to properly address executive functions.

We showed that wild-type adolescent mice formed an attentional set, as demonstrated by the increased number of trials to complete the EDS. Newman and colleagues found similar results, using a manual “digging” version of the task in rats, where they required fewer trials to reach criterion on the ID than the CD but more trials to reach criterion on the EDS than IDS (Newman and McGaughy, 2011). Similarly, Cain et al. (2011) as well as Shepard et al. (2017) reported that adolescent rats and mice need more trials to complete EDS than IDS. Thus, our findings are in agreement with these previous studies, supporting the evidence of an already established ability to develop an attentional set during adolescence in mice. Early detection and early intervention could be potentially more effective in mitigating or reversing the pathological trajectories and ultimately the life quality of individuals with cognitive deficits (Elvevåg and Goldberg, 2000; Hintze and Borkowska, 2015). In this context, our task of assessing executive functions in mice could allow us to test early interventions and early therapy strategies, strictly controlling environmental and genetic factors. Furthermore, our automatic procedure provides some advantage in the application of optogenetics and electrophysiological measurements (for example), as well as in large screenings of pharmacological and/or genetic effects.

In contrast to adults, we were not able to detect any increased effort in solving reversal learning stages in adolescent mice. This is in agreement with previous studies in mice reporting no effects in the reversal stages (Shepard et al., 2017). Moreover, in agreement with our findings, it has been observed that under conditions of enhanced choice uncertainty, juvenile mice required fewer trials to complete the reversal phase, showing more rapid reversal learning than adults (Johnson and Wilbrecht, 2011). In contrast, two studies in rats reported that adolescent rats required more trials to complete the reversal phase of a discrimination, in particular when performing the reversal for the first time (Cain et al., 2011; Newman and McGaughy, 2011). This might suggest species differences related to reversal learning between rats and mice. Nevertheless, reversal learning requires inhibition of an established response as well as the acquisition of a new response (Remmelink et al., 2016). Thus, our results on reversal learning might suggest two scenarios: (i) adolescent mice performed each stage separately, as a blank page; (ii) adolescent mice adapted more efficiently their responses to varying reward contingencies. The first scenario is contested by the finding that mice required more trials to solve the EDS, indicating the formation of a cognitive set of attention. Conversely, the hypothesis that adolescent mice were more efficient in adapting their responses during reversal learning, but not on the EDS, would confirm that this task assessed distinct cognitive constructs, subserved by different brain regions (Bissonette et al., 2008; Keeler and Robbins, 2011).

To further validate this task, we tested the dysbindin1-dependent alterations on the maturation of higher-order cognitive functions from adolescence to adulthood. In agreement with adult mice, a reduced number of DYS1 knockout mice were able to complete the entire task, suggesting early alterations in executive functions’ abilities. However, here we found that DYS1 knockout adolescent mice had a more prominent deficit in solving the IDS stage, while no effect was detectable in the EDS stage. This is in contrast to adult mice, in which we found a selective DYS1-dependent deficit in the EDS stage (Scheggia et al., 2018). Adolescents are reported to respond relatively more prominently to external than internal stimuli compared to adults (Ernst et al., 2011). Moreover, the inhibitory control towards irrelevant stimuli is still not well developed during adolescence (Brocki and Bohlin, 2004). Thus, this might suggest that the deficits found in the IDS stage in adolescent DYS1 knockout mice could be related to a delayed maturation of inhibitory control towards external irrelevant stimuli, such as those presented during the first IDS stage.

Overall, our study provides a new tool to test the developmental trajectories of executive functions in mice, with the possibility to run genetics, pharmacological and mechanistic studies during the adolescent period.

## Data Availability Statement

The raw data supporting the conclusions of this article will be made available by the authors, without undue reservation.

## Ethics Statement

The animal study and all procedures were reviewed and approved by the Italian Ministry of Health (permit n. 230/2009-B) and strictly adhere to the recommendations in the Guide for the Care and Use of Laboratory Animals of the National Institutes of Health.

## Author Contributions

MC and FP designed the study. MC and DS performed the behavioral tests and the statistical analyses. All authors contributed to discussions about the results and critically revised the manuscript. All authors contributed to the article and approved the submitted version.

## Conflict of Interest

The authors declare that the research was conducted in the absence of any commercial or financial relationships that could be construed as a potential conflict of interest.
